# Changes in primary metabolites and volatile organic compounds in cotton seedling leaves exposed to silver ions and silver nanoparticles revealed by metabolomic analysis

**DOI:** 10.7717/peerj.13336

**Published:** 2022-04-21

**Authors:** Yong Yang, PengMeng Du, Wenjie Lai, Liyan Yin, Yuanhao Ding, Zhonghua Li, Haiyan Hu

**Affiliations:** Hainan Key Laboratory for Sustainable Utilization of Tropical Bioresources, College of Tropical Crops, Hainan University, Haikou, Hainan, China

**Keywords:** Cotton, Primary metabolites, Volatile organic compounds, Silver nanoparticles

## Abstract

In the area of climate change, nanotechnology provides handy tools for improving crop production and assuring sustainability in global agricultural system. Due to excellent physiological and biochemical properties, silver nanoparticles (AgNPs) have been widely studied for potential use in agriculture. However, there are concerns about the mechanism of the toxic effects of the accumulation of AgNPs on crop growth and development. In this study, the impacts of AgNPs on cotton (*Gossypium hirsutum*) seedlings were evaluated by integrating physiological and comprehensive metabolomic analyses. Potting-soil-grown, two-week-old cotton seedlings were foliar-exposed to 5 mg/plant AgNP or 0.02 mg/plant Ag^+^ (equivalent to the free Ag^+^ released from AgNPs). Primary metabolites and volatile organic compounds (VOCs) were identified by gas chromatography–mass spectrometry (GC-MS) and solid-phase microextraction (SPME) GC-MS, respectively. AgNPs inhibited the photosynthetic capacity of the cotton leaves. The metabolic spectrum analysis identified and quantified 73 primary metabolites and 45 VOCs in cotton leaves. Both treatments significantly changed the metabolite profiles of plant leaves. Among the primary metabolites, AgNPs induced marked changes in amino acids, sugars and sugar alcohols. Among the VOCs, 13 volatiles, mainly aldehydes, alkanes and terpenoids, were specifically altered only in response to AgNPs. In summary, our study showed that the comprehensive influence of AgNPs on primary metabolites and VOCs was not merely attributed to the released Ag^+^ but was caused by AgNP-specific effects on cotton leaves. These results provide important knowledge about the physiological and chemical changes in cotton leaves upon exposure to AgNPs and offer a new insight for supporting the sustainable use of AgNPs in agriculture.

## Introduction

Engineered nanomaterials offer a promising alternative for crop disease management and provide potential advantages with high efficacy and lower ecotoxicity (Elmer, Ma & White, 2018; Muthukrishnan, Murugan & Selvaraj, 2019). Nanoparticles were developed as plant-growth stimulators and fungicides to prevent fungal diseases or were applied as agents to enhance fruit ripening (Castillo-Henríquez et al., 2020; Monica & Cremonini, 2009; Steinitz & Bilavendran, 2011; Vinković et al., 2017). Among the various types of nanoparticles, silver nanoparticles (AgNPs) are the most widely applied nanomaterial (Wijnhoven et al., 2009). In the agricultural sector, the outstanding properties of AgNPs are of great interest to researchers developing suitable antimicrobial agents (Acharya & Pal, 2020; Chandra et al., 2020; Menazea & Ahmed, 2020; Panicker et al., 2020). AgNPs could inhibit the infection of pathogenic bacteria and promote plant survival (Ali et al., 2015; Ibrahim et al., 2020; Narayanan & Park, 2014). Although AgNPs have tremendous potential and beneficial impacts in a wide range of applications, the risks associated with their uses should still be thoroughly evaluated (Fadeel et al., 2018).

In recent years, the toxicity of AgNPs to plants has been reported in a number of studies. The growth of mustard seedlings declined as a result of inhibition of photosynthesis after AgNP treatment (Vishwakarma et al., 2017). AgNPs suppressed the growth of pearl millet seedlings by accelerating reactive oxygen species production and changing their membrane permeability (Khan et al., 2019). AgNPs induced a decrease in photosynthetic activity and chlorophyll content and altered the chloroplast ultrastructure of tobacco plants (Peharec Štefanić et al., 2021).

Most of these studies have focused on the effects of AgNPs at physiological and biochemical levels. The development of broad profiling approaches, such as genomics, transcriptomics and metabolomics, has aided the exploration of the diversity of plant metabolism and the underlying molecular mechanisms by which plants control their chemical composition (Fernie & Tohge, 2017). It has been reported that the transcriptome profile changes in *Arabidopsis thaliana* show that genes involved in photosynthesis are altered in response to AgNPs (Zhang et al., 2019). Changes in the rice proteome under AgNP stress were found to be involved in oxidative stress tolerance, cell wall, direct DNA/RNA/protein damage, cell division and apoptosis (Mirzajani et al., 2014). Moreover, metabolomic analysis showed the toxicity and detoxification mechanisms of AgNPs on cucumbers (Zhang et al., 2018a). Therefore, omics technology allows us to explore the genetic and molecular changes underlying crop responses to AgNPs, which will be highly informative for their sustainable application as crop protection products.

Low molecular weight metabolites can provide a readout of the physiological status and a bridge between the genotype and the phenotype (Sanchez et al., 2008; Yang et al., 2017). Metabolomics has aided in exploring the identification and quantification of the thousands of small molecules by which plant cells control their chemical composition (Fang, Fernie & Luo, 2019). Plant volatile organic compounds (VOCs) are typically lipophilic molecules with a high vapor pressure and they play critical roles in plant interactions with their environment to ensure protection from pathogens and pests (Dudareva et al., 2013; Pichersky, Noel & Dudareva, 2006). Volatilomics plays an important role in fundamental plant biology and applied biotechnology (Zeng et al., 2017; Zu et al., 2020).

Cotton (*Gossypium hirsutum*) is the most important natural fiber crop in the world and is cultivated in more than 75 countries (Chen et al., 2007). Cotton can be grown in areas contaminated by heavy metals, both for income and for soil restoration. To elucidate whether the effects of AgNPs originate from Ag^+^ or from nanoparticles and assess AgNP toxicity, we investigated the impact of AgNPs and Ag^+^ on the leaf accumulation of primary and volatile metabolites by using gas chromatography–mass spectrometry (GC-MS) and solid-phase microextraction (SPME) GC-MS techniques. The objectives of this research were to investigate AgNP effects on growth and metabolic response of cotton seedlings and provide valuable information for the risk assessment of AgNPs in agriculture.

## Materials & Methods

### Characterization of AgNPs

AgNPs (polyvinyl pyrrolidone coated) were synthesized using previously described methods (Cheng et al., 2011; Zhang et al., 2019). Before each exposure, the AgNP suspensions were sonicated at 45 kHz for 10 min to obtain a well-dispersed solution using an ultrasonic bath (WD-9415B, Beijing, China). The actual size distribution and morphology of the AgNPs were identified by transmission electron microscopy (TEM, Hitachi, Japan). Silver nitrate solution (AgNO_3_) was purchased from Sigma Aldrich.

### Plant culture and exposure

Experiments were carried out during April 2021 in a greenhouse at Hainan University, Haikou, China. The cotton seeds *Gossypium hirsutum* cv. TM-1 used in this study were immersed in deionized water for 48 h before germination in the dark and then cultivated in the potting soil (Pindstrup, Denmark). The cotton seedlings were grown in the greenhouse (28–35 °C by day and 20–25 °C by night) under a 16/8 h light/dark cycle. Foliar exposure was initiated when the cotton seedlings were 2 weeks old. Three treatments were established, including control (CK, ultrapure water), Ag^+^ (AgNO_3_) and AgNP treatments. Our previous study showed that approximately 0.4% Ag^+^ was released from AgNPs (Zhang et al., 2019). Therefore, 0.02 mg Ag^+^ was set up in paralleled to 5 mg AgNPs per plant treatment. Fifteen replicate plants (one plant per pot) were obtained for each treatment. Stock solutions of 100 mg AgNPs per liter and 0.4 mg of AgNO_3_ per liter were prepared in ultrapure water, as this dose of AgNPs previously showed effects on virus acquisition and acted as a fungicide on wilt disease with no negative impacts on plants or the soil community (El Gamal et al., 2022; Kaur et al., 2018). The application was performed three times per day for a week by using a hand-held spray bottle, with a total volume of 50 mL per plant during 7 days of exposure, resulting in an approximate total delivered mass of 0.02 mg Ag^+^ per plant and 5 mg of AgNPs per plant. The treated cotton leaves were collected and frozen in liquid nitrogen immediately and stored at −80 °C until use for metabolite analysis.

### Physiological and photosynthetic parameter analysis

After 7 days of exposure, the cotton seedlings were thoroughly washed with deionized water to remove any residual particles. The seedlings were separated into leaves, stems and roots for weighing. Chlorophyll was extracted using 10 mL of 95% ethanol in 15 mL tubes. The tubes were placed in the dark for 24 h until all of the leaves became chlorotic. The absorbance was measured at 665 nm, 649 nm and 470 nm using 95% ethanol as a control. The concentrations of chlorophyll a and b and carotenoids were calculated as previously described (Lichtenthaler & Wellburn, 1983): chlorophyll *a* = 13.36 × A665 − 5.19 × A649, chlorophyll *b* = 27.43 × A649 − 8.12 × A665, carotenoids = (1,000 × A470 − 2.05 × chlorophyll a − 114.8 × chlorophyll b)/245.

Plant photosynthetic parameters were measured with a Li-6800 portable gas-exchange system (Li-Cor, Lincoln, Nebraska, USA). The measurement parameters included the net photosynthetic rate (Pn), stomatal conductance (Gs), intercellular CO_2_ concentration (Ci) and transpiration rate (Tr). Manual control conditions were applied at a CO_2_ flow rate of 400 µmol s^−1^ and at a photosynthetic photon flux density of 1,000 µmol m^−2^ s^−1^. The chlorophyll fluorescence parameters, such as the quantum yield of nonregulated energy dissipation Y(NO), nonphotochemical quenching Y(NPQ) and the measure of overall reduced and oxidizable PSII centers (qP), were used as indicators of the photosystem efficiency (Kramer et al., 2004). Chlorophyll fluorescence characteristics were determined using a PAM-2500 portable chlorophyll fluorescence apparatus (PAM-2500; Walz, Germany). The leaves were adapted to dark conditions for 30 min before the measurement.

### Metabolite and VOC extraction

Frozen leaves were ground into powder in a cryogenic mill and 0.1 g of tissue was transferred to a two mL microcentrifuge tube. Then, one mL of 70% methanol was added to each sample while vortexing the tubes. The samples were extracted for 12 h at 4 °C and centrifuged at 12,000 rpm for 5 min. The supernatant was transferred to a two mL tube and vortexed for 4∼6 h until all of the methanol evaporated. Then, 100 µL of 20 mg/mL methoxyamine pyridine solution was added to the dried residue. The mixture was vortexed vigorously until it completely dissolved, and then it was heated at 37 °C for 1 h. The extracts were derivatized by adding 100 µL N-methyl-N-(trimethylsilyl)-trifluoroacetamide (MSTFA, Sigma–Aldrich, USA) and heating at 60 °C for 3 h. Quality control samples were prepared by mixing aliquots of each sample and analyzing them every 9 samples to evaluate the analytical validation.

A total of 0.5 g powder was weighed and transferred into a 22 mL glass headspace vial (Agilent Technologies, Inc.), incubated for 10 min at 37 °C. Then, 1 g CaCl_2_⋅2H_2_O and one mL of 100 mM EDTA-NaOH (Sinopharm Chemical Reagent Co., Ltd., Shanghai, China) (pH 7.5) were added and mixed thoroughly by sonication for 5 min. The VOCs were absorbed onto a one cm 50/30 µm SPME fiber with DVA/CWR/PDMS (divinylbenzene/carbon wide range/polydimethylsiloxane) (Supelco, http://www.supelco.com/). After a 20 min adsorption period at 50 °C while shaking, the samples were desorbed for 2 min at 270 °C in GC–MS. Quality control samples were injected every 9 samples throughout the analytical run.

### GC-MS and chemical analysis

The metabolites and VOCs were detected on a gas chromatography instrument (7890A GC, Agilent Technologies, Santa Clara, CA, USA) equipped with an Agilent 7000 mass spectrometer. The HP-5 MS capillary column (Agilent Technologies, Santa Clara, CA, USA) with a 30 m length 0.25 mm diameter and 0.25 µm film thickness was used to separate VOCs. The temperature program was as follows: 40 °C for 3 min and then increasing to 160 °C at 2 °C/min. Subsequently, the temperature was ramped at 50 °C/min to 300 °C and held for 3 min. The injector temperature was set at 270 °C. Helium was used as the carrier gas at a flow rate of one mL/min. The mass spectra were scanned at 50–650 m/Z. The metabolites were analyzed using the derivatized samples. The initial temperature column oven was set at 70 °C for 3 min, increased to 300 ° C at 10 °C/min and kept at 300 °C for 5 min. The injection volume was set to 1 µL in spitless mode. The temperatures of the injector, MS quadrupole and ion source were kept at 270 °C, 150 °C and 230 °C. The electron collision energy was set at 70 eV. The data were acquired with the full scan mass range (*m/z* 50 to 650), and the solvent delay time was 5.4 min.

To identify the separated compounds, the GC-MS raw data were compared to the chemical structures in the National Institute of Standards and Technology NIST library (Version 2.4) database by using the MassHunter Qualitative Analysis Navigator (Agilent Technologies, Santa Clara, CA, USA). The identified compounds were then detected based on the retention index (RI) and mass spectra. The peak areas of the mass spectra signal were used to calculate the relative contents of the components by using MassHunter Quantitative Analysis (Agilent Technologies, Santa Clara, CA, USA). All of these analyses were performed in triplicate.

### Statistical analysis

Unsupervised principal component analysis (PCA) and biological pathway analysis were performed on GC-MS data via an online server (https://www.metaboanalyst.ca/) (Pang et al., 2021). Before PCA, the data were normalized by data scaling to make the individual features more comparable. The statistical significance of the metabolite and VOC data was determined using the Student’s *t-test* at *p* < 0.05 (*n* = 3). The impact value of the pathway enrichment analysis was set at 0.1 (Xia & Wishart, 2010).

The data for each physiological and biochemical treatment were analyzed by SPSS 26.0 (IBM, NY, USA). An analysis of variance (ANOVA) test was used to determine the treatment effects on the measured variables. Post hoc Duncan’s multiple range test was performed to compare the means at *p* < 0.05.

## Results

### AgNP characterization

**Figure 1:**
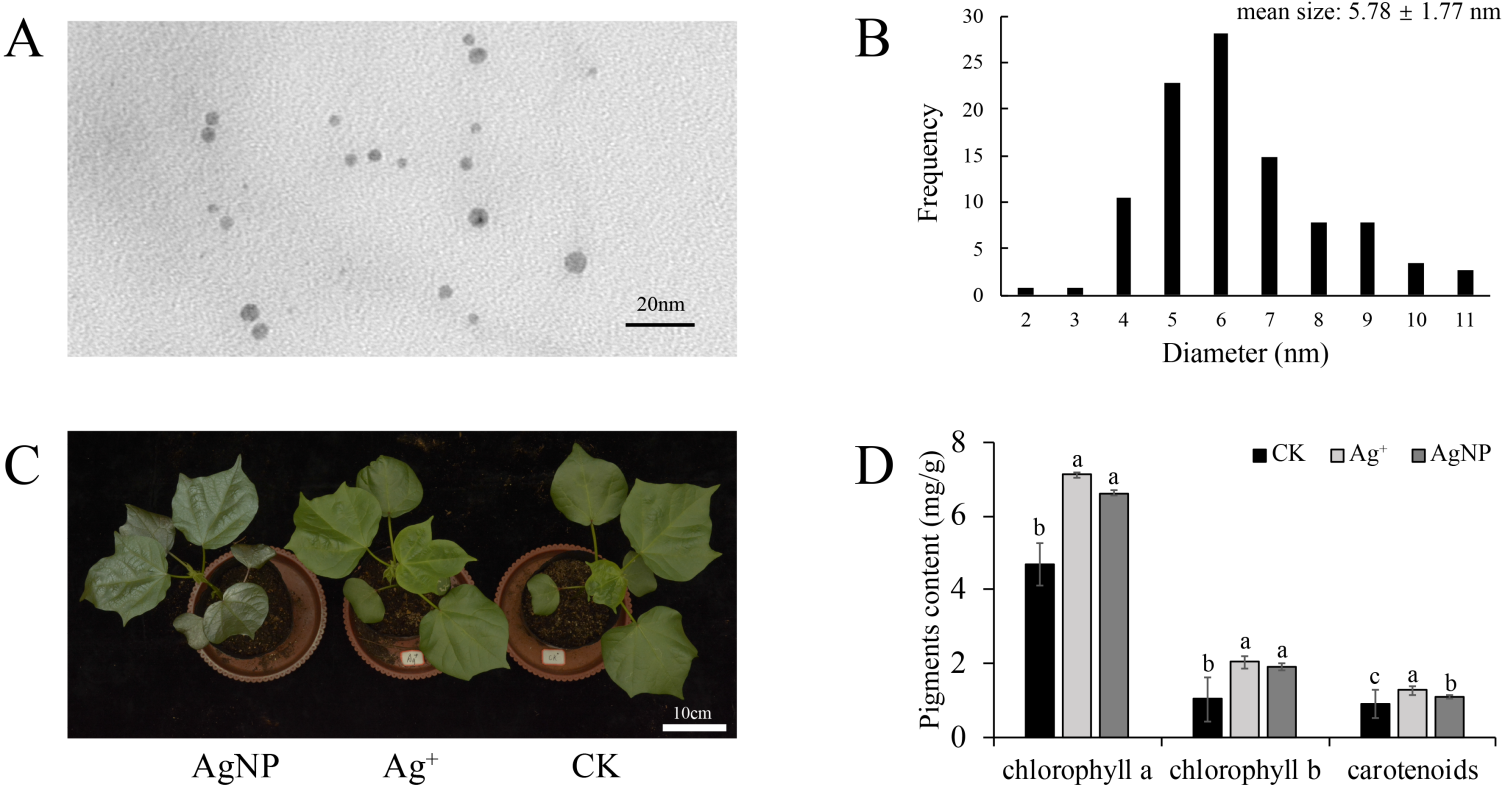
Morphology of the nanoparticles. (A & B) Transmission electron microscopy (TEM) images and frequency of the different AgNP sizes, (C & D) phenotypes and photosynthetic pigment contents of the cotton seedlings after exposure to Ag^+^ and AgNPs for one week (CK, ultrapure water exposure plants; Ag^+^, AgNO_3_ exposure plants; AgNP, AgNP exposure plants). Error bars represent the standard error of three biological replicates. The same letters represent no significant differences.

To confirm nanoparticle morphological characteristics, the AgNP solution was analyzed with TEM. It showed that the AgNPs were spherical and uniformly dispersed (Fig. 1A). The average diameter of the AgNPs was 5.78 ± 1.77 nm as measured by ImageJ software (Fig. 1B). It is believed that AgNPs of 5 nm demonstrate the best bactericidal activity and are internalized by cells (Agnihotri, Mukherji & Mukherji, 2014). Previously, we demonstrated that AgNPs could be transferred in *A. thaliana* and accumulate in leaves (Zhang et al., 2019). Thus, these AgNPs were suitable for our treatments.

### Phenotypes, biomass and photosynthetic pigment content

To explore the physiological responses of cotton seedlings under AgNP exposure, the effects of AgNPs and Ag^+^ on phenotype, biomass and photosynthetic pigments were analyzed. After one week of exposure, none of the treatments showed significant toxicity symptoms, only exhibiting a darker leaf color under AgNP treatment due to its original color (Fig. 1C). The contents of photosynthetic pigments were significantly changed in both the Ag^+^ and AgNP groups. The Ag^+^ and AgNP treatment resulted in a significant increase in chlorophyll a (*p* = 0.003, 0.009) and b (*p* = 0.000, 0.001) and carotenoid contents (*p* = 0.003, 0.042) (Fig. 1D). However, the root, stem and leaf biomass of the cotton seedlings were not significantly impacted after exposure to 0.4 ppm Ag^+^ or 100 ppm AgNPs for 1 week (Figs. S1A, S1B and S1C). It has been reported that the application of Ag^+^ and AgNPs did not significantly increase the biomass of cucumbers after one week (Zhang et al., 2018a). This result indicated that one week of treatment was not enough to change the biomass of cotton seedlings.

### Photosynthetic capacity and chlorophyll fluorescence

**Figure 2:**
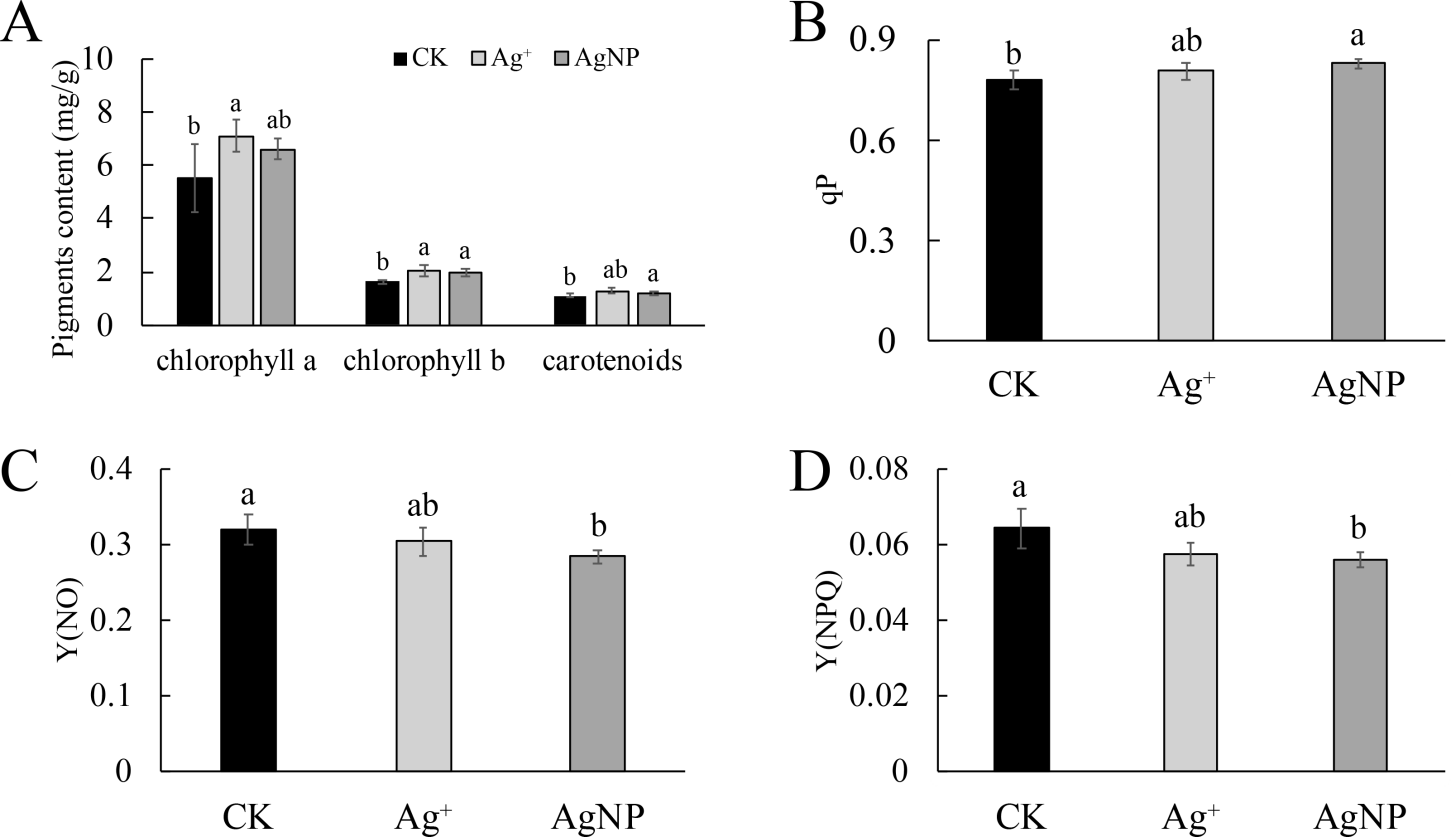
Photosynthetic parameters under Ag^+^ and AgNP treatments. (A) Net photosynthetic rate (Pn), (B) coefficient of photochemical quenching (qP), (C) yield of nonregulated heat dissipation of PSII (Y(NO)) and (D) effective quantum yield of regulated nonphotochemical quenching (Y(NPQ)) of cotton leaves after Ag^+^ and AgNP treatments. Error bars represent the standard error of three biological replicates, and the same letters represent for no significant differences.

As the photosynthetic pigments changed, their photosynthetic capacity was comprehensively detected. Treatment with AgNPs exhibited stronger effects on their photosynthetic capacity than Ag^+^. Namely, the net photosynthetic rate (Pn) was significantly (*p* = 0) decreased under AgNP treatment (Fig. 2A), while the intercellular CO_2_ concentration (Ci), transpiration rate (Tr) and stomatal conductance (Gs) were not significantly changed in response to either Ag^+^ or AgNP treatment (Figs. S2A, S2B and S2C). In addition, the photochemical qP increased significantly under AgNP treatment (*p* = 0.038), while nonphotochemical quenching [Y(NPQ)] (*p* = 0.035) and the quantum yield of nonregulated energy dissipation [Y(NO)] (*p* = 0.047) decreased (Figs. 2A, 2B and 2C).

### Primary metabolite response to AgNPs

**Figure 3:**
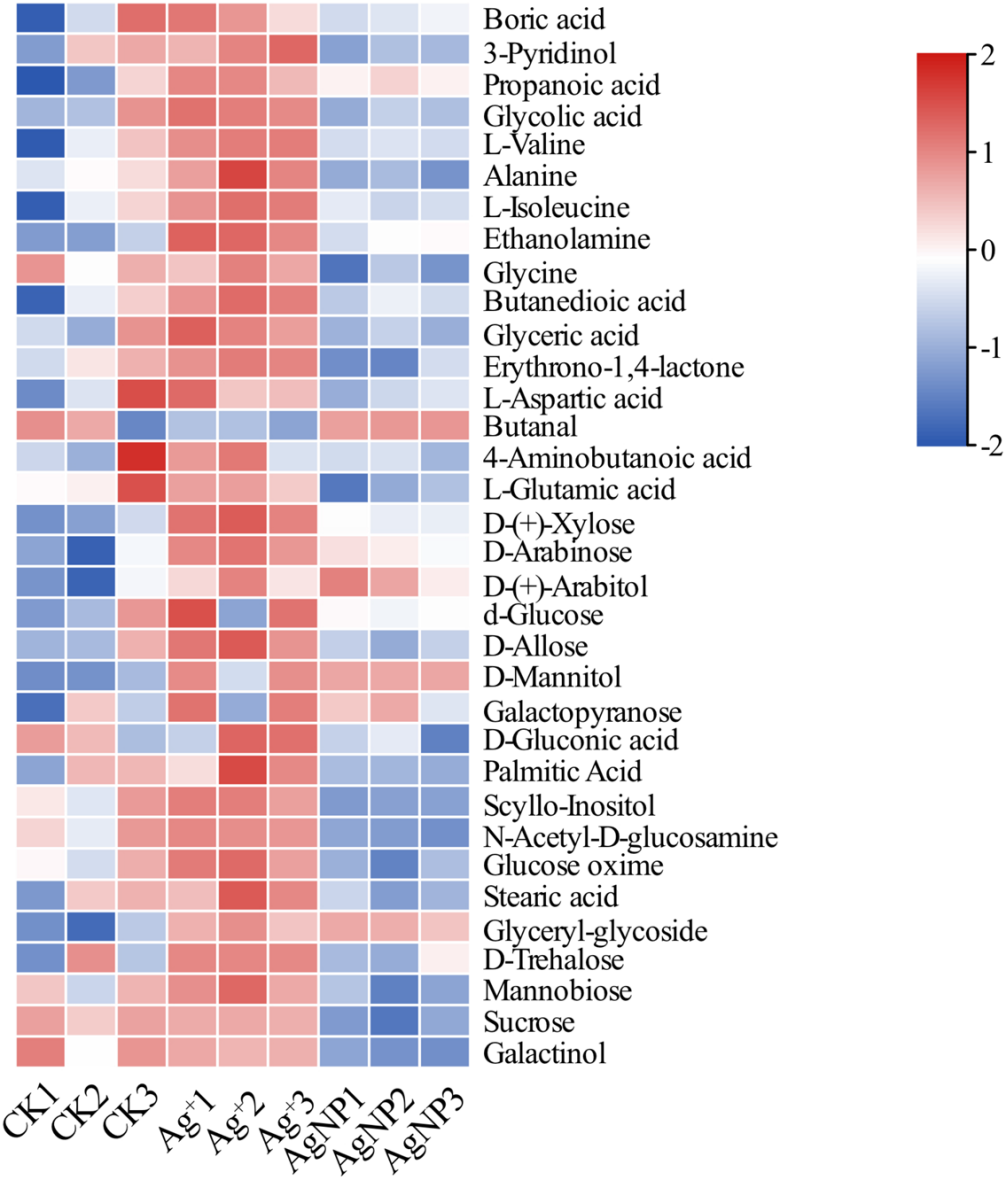
Heatmap visualization of 34 significantly differential metabolites in the samples. Heatmap showing the significantly differential metabolites after Ag^+^ and AgNP treatments (red and blue colors indicate relatively high and low metabolite contents, respectively).

Due to their special physiochemical characteristics, AgNPs may be involved in key metabolic progresses. To further determine the metabolite changes in cotton leaves, nontargeted GC-MS metabolomics was conducted to identify the relative quantitation of primary metabolites. In total, 73 metabolites were identified and quantified in cotton leaves after exposure to 0.4 ppm Ag^+^ or 100 ppm AgNPs (Table S1). First, PCA was performed to overview the similarities between samples. The PCA score plot showed that the 3 groups were clustered along the PC1 and PC2, which explained 52.2% and 15.5% of the total variance, respectively (Fig. S3). To gain insight into the metabolite response to Ag^+^ and AgNPs, we identified significantly differential metabolites in Ag^+^ and AgNP samples in comparison with CK samples. The relative content of these significantly differential metabolites is shown in Fig. 3.

**Figure 4:**
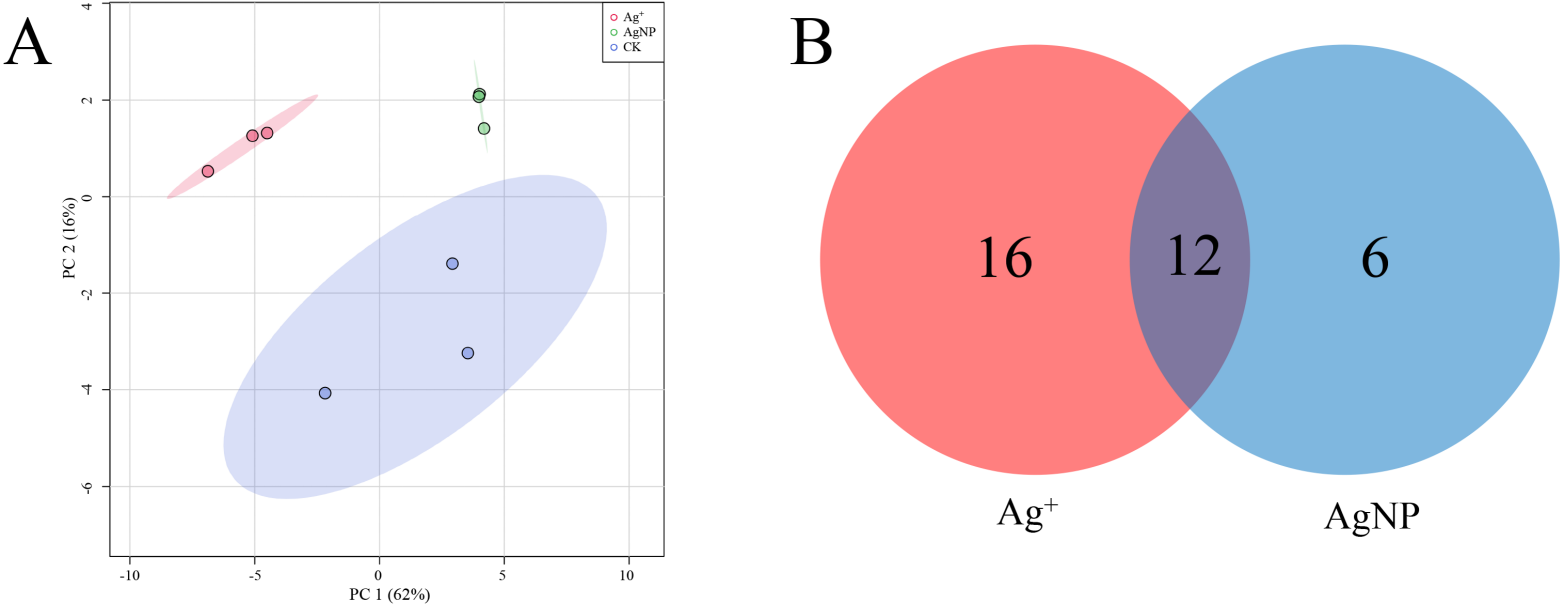
Characterization of significantly differential metabolites in all the samples. (A) PCA score plots of specific differential metabolite profiles in cotton leaves after Ag^+^ and AgNP treatments. (B) Venn diagram depicting the specific differential metabolites in the Ag^+^ and AgNP treatment groups.

PCA of the significantly differential metabolites exposed to Ag^+^ and AgNPs indicated high reproducibility of the biological replicate samples. The different groups were clearly separated, with a total variance of 78%, which showed that the 3 groups were easily discriminated (Fig. 4A). Furthermore, Venn diagram analysis showed that treatment with Ag^+^ and AgNPs caused 28 and 18 metabolites, respectively, to be significantly changed (Fig. 4B). Specifically, treatment with Ag^+^ induced upregulation of 25 metabolites and downregulation of 3 metabolites, while treatment with AgNPs induced upregulation of 7 metabolites and downregulation of 11 metabolites (Fig. S4). These results demonstrated that more primary metabolites changed in response to Ag^+^ treatment than AgNPs in cotton leaves. Previous studies showed that ionic silver induced more toxic effects than AgNPs in green algae. Low concertation Ag^+^ induced more significantly changed metabolites than AgNPs in N_2_-fixing cyanobacteria (Huang et al., 2020). In this experiment, the changes in the metabolic profiles of the Ag^+^ group was greater than that in the AgNP group, which may be because Ag^+^ from AgNO_3_ is more available than Ag^+^ from AgNPs.

Additionally, 12 primary metabolites (alanine, propanoic acid, ethanolamine, glutamic acid, xylose, arabitol, mannitol, galactopyranose, gluconic acid, glyceryl-glycoside, sucrose and galactinol) showed significant changes, which were common in Ag^+^- and AgNP-treated leaves. Six primary metabolites (3-pyridinol, glycine, palmitic acid, acetyl-glucosamine, stearic acid and mannobiose) were specifically changed in response to AgNPs only (Fig. S5). Biological pathway analysis revealed that significantly affected primary metabolites in AgNP-exposed leaves were mainly associated with alanine, aspartate and glutamate metabolism, galactose metabolism, glyoxylate and dicarboxylate metabolism, glutathione metabolism, ascorbate and aldarate metabolism, and glycine, serine and threonine metabolism (Fig. S6).

### VOC response to AgNPs

**Figure 5:**
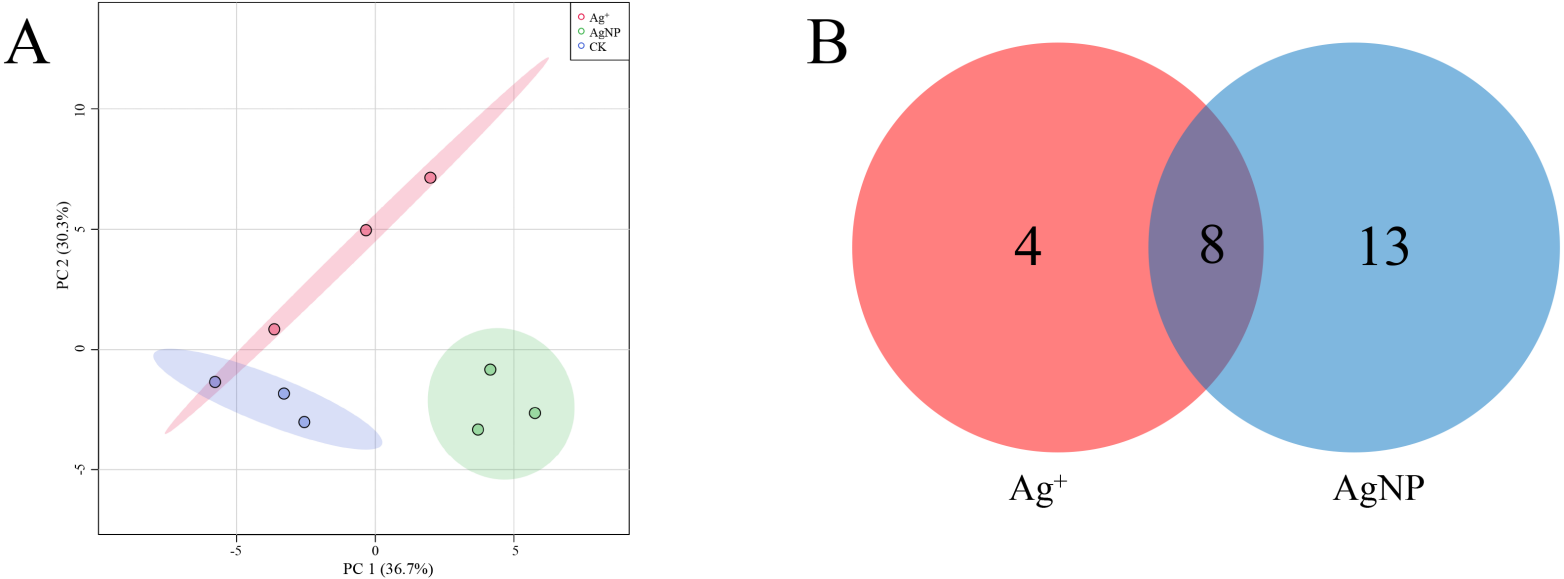
Characterization of significantly differential VOCs in the samples. (A) PCA score plots of VOC profiles in cotton leaves. (B) Venn diagram depicting the specific differential metabolites in the Ag^+^ and AgNP treatment groups.

The SPME GC-MS technique was used to identify VOCs in cotton leaves. A total of 45 VOCs were identified and quantified. In this study, the identified VOCs were mainly lipids, lipid-like compounds, benzenoids and organic oxygen compounds (Table S2). To characterize the overall differences in VOCs, the PCA of all samples were classified by similarity. Two principal components cumulatively accounted for 67% of the total variation, with PC1 explaining 36.7% and PC2 explaining 30.3% of the variance (Fig. 5A). The replicate samples of each group were clustered together, and the CK, Ag^+^ and AgNP groups were partly discriminated. To better understand the response of the cotton leaves to 0.4 mg/L Ag^+^ or 100 mg/L AgNPs, significantly differential VOCs were screened out. Specifically, 12 and 21 significantly different VOCs were identified in the Ag^+^ and AgNP treatment groups, respectively. Eight VOCs were significantly changed in response to both treatments (Fig. 5B). Thus, AgNPs induced significant differences in a higher number of VOCs than Ag^+^.

**Figure 6:**
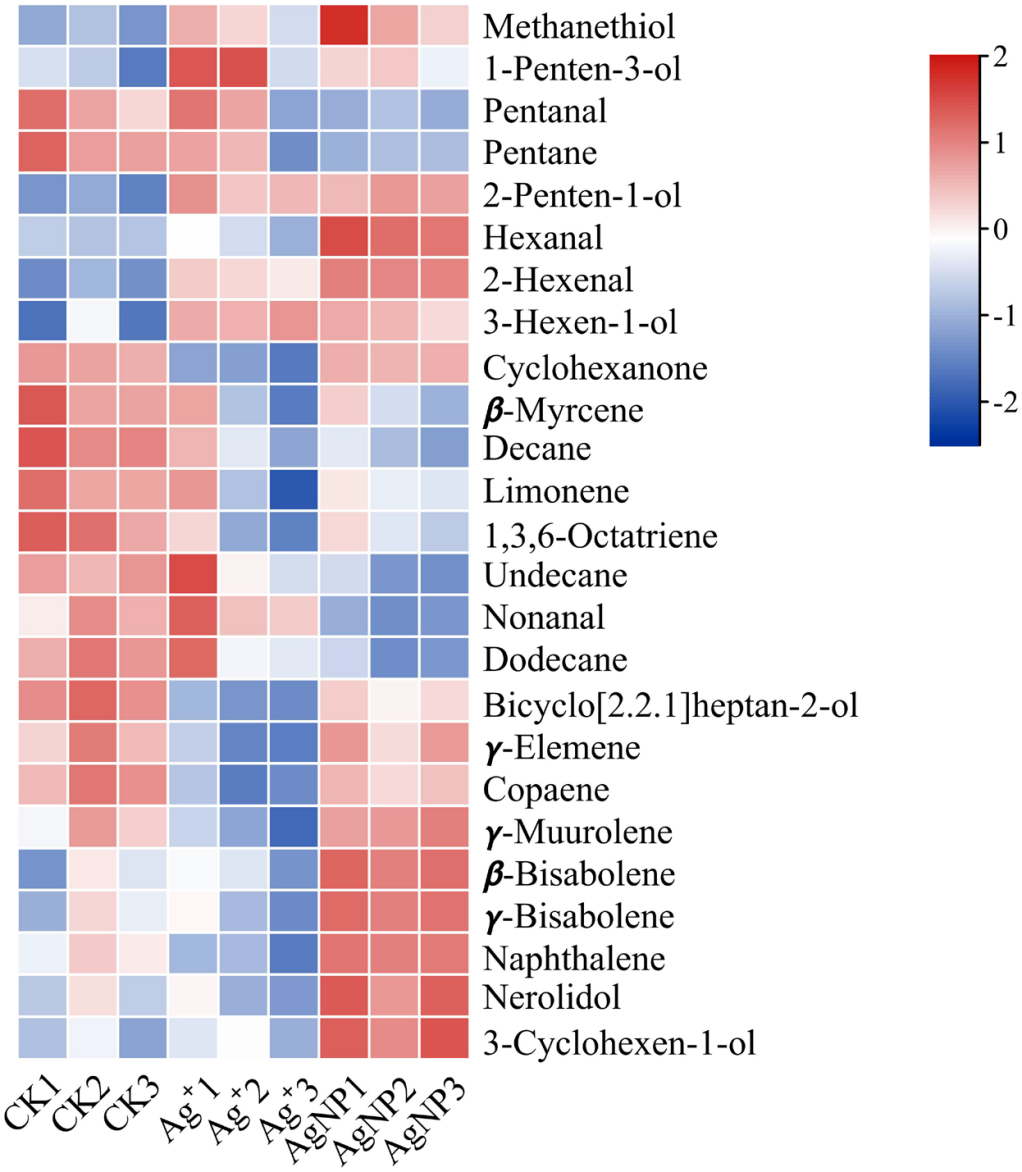
Heatmap visualization of 25 significantly differential VOCs in the samples. The heatmap shows the significantly differential VOCs after Ag^+^ and AgNP treatments (red and blue colors indicate relatively high and low metabolite contents, respectively).

In addition, four (methanethiol, 2-penten-1-ol, 2-hexenal, 3-hexen-1-ol) and 11 (methanethiol, 2-penten-1-ol, 2-hexenal, 3-hexen-1-ol, 1-Penten-3-ol, hexanal, *β*-bisabolene, *γ*-bisabolene, nerolidol, 3-cyclohexen-1-ol, naphthalene) VOCs were upregulated and 8 (decane, 1,3,6-octatriene, bicyclo[2.2.1]heptan-2-ol, naphthalene, cyclohexanone, *γ*-elemene, copaene, *γ*-muurolene) and 10 (decane, 1,3,6-octatriene, bicyclo[2.2.1]heptan-2-ol, pentanal, pentane, *β*-myrcene, limonene, undecane, nonanal and dodecane) VOCs were down-regulated in the Ag^+^ and AgNP treatment groups, respectively. A heatmap was generated using the relative abundances of the 25 VOCs (Fig. 6). The significantly differential VOCs from Ag^+^ or AgNP treatment were selected for PCA. The different groups were clearly separated, with a total variance of 82.2%, which showed that the groups were easily discriminated (Fig. S7). These results implied that the toxicity of AgNPs not only resulted from the free Ag^+^ released from nanoparticles, but was also affected by the nanoparticles. Therefore, theses 25 VOCs are expected to be the new hallmarks of AgNP exposure.

## Discussion

In this study, Ag^+^ or AgNPs was applied to cotton seedling leaves. The growth of cotton seedlings showed no significantly differences under Ag^+^ or AgNP treatment, but the phenotype and photosynthesis of cotton seedlings were much more influenced under AgNP treatment. Previous studies reported that the chlorophyll content of *Hydrilla verticillata* increases after AgNP and AgNO_3_ treatment (Jiang et al., 2017a). In our data, the pigment contents were increased in both Ag^+^ and AgNP treatments, indicating that chlorophyll changes resulted from the release of Ag^+^ rather than the nanoparticles. Moreover, a high value of Y(NPQ) indicates a high photoprotective capacity, while Y(NO) reflects the closure of reaction centers of PSII and the excess of energy being passively dissipated in the form of heat and fluorescence (Deng et al., 2013; Jiang et al., 2017b). A lower measure of overall reduced and oxidizable PSII center (qP) values resulted in a decrease in the plant chlorophyll content (Khan et al., 2019; Vishwakarma et al., 2017). It can also be seen in tobacco that AgNPs induce a greater more inhibitory effect on photosynthesis than Ag^+^ (Peharec Štefanić et al., 2021). Carotenoids are involved in the NPQ of excess energy to prevent photoinhibition and photodamage. Our results demonstrated that an increase in the qP resulted in an increased chlorophyll b content to lower NPQ and NO to maintain the photosynthesis. Therefore, these results indicated that the AgNPs inhibited the photosynthetic capacity of cotton seedlings.

The metabolites of plants were dramatically affected by environmental stimuli. Glycine and serine are two important amino acids formed during photorespiration, and they act as indicators of photorespiratory activity (Diaz et al., 2005). Photorespiration may be protected under high light-induced stress by supplying additional glycine (Osmond et al., 2000). Glycine was significantly decreased when plants were exposed to AgNPs and a derivative of serine was significantly increased, suggesting that photorespiration was inhibited. Sugars are important signaling molecules involved in the adaption to abiotic stress (Sami et al., 2016). Low concentrations of glucose and sucrose function as osmotic adjustments and strengthen the carbon energy reserves under salt stress (Boriboonkaset et al., 2013; Hu et al., 2012). A significant increase in the accumulation of xylose, arabitol, mannitol, galactopyranose and glyceryl-glycoside was identified upon exposure to AgNPs, while alanine, glutamic acid, gluconic acid, pyridinol, glycine, palmitic acid, acetyl-glucosamine, sucrose, mannobinose and galactinol were significantly decreased (Fig. S5). Among them, pyridinol, glycine, palmitic acid, glucosamine, stearic acid and mannobiose were specially altered in the AgNP treatment. In summary, the changes in glycine, sugars and sugar alcohols might indicate perturbations in carbon and nitrogen metabolism to manage plant development to stimulate defense responses upon AgNP exposure.

**Figure 7:**
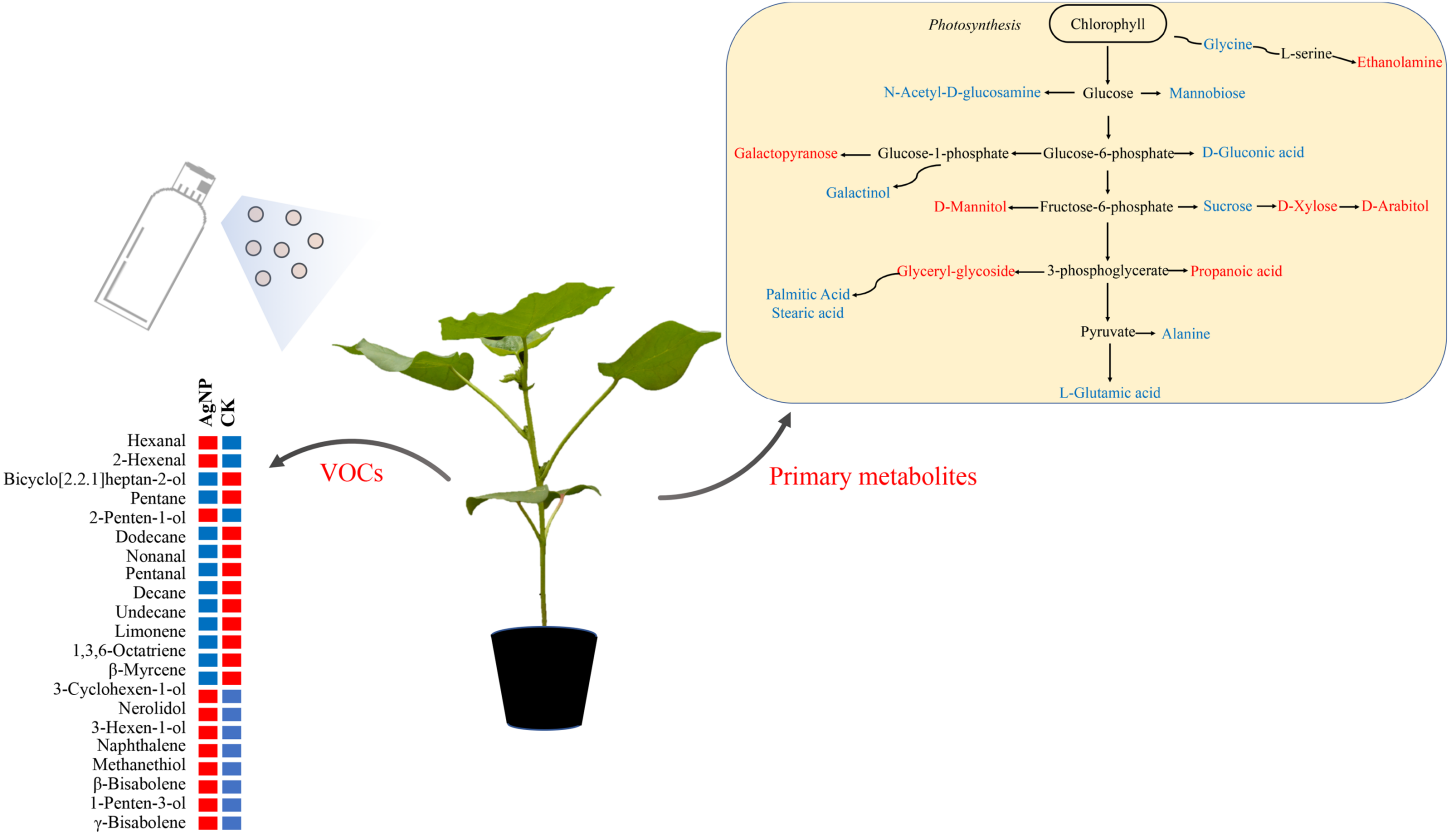
Schematic diagram of the proposed metabolic pathway and VOCs in cotton leaves exposed to AgNPs. Red and blue (right panel) represent up- and downregulated in primary metabolites in the AgNP group compared to the CK group. Red and blue (left panel) indicate the relatively high and low content of the significantly differential VOCs in the AgNP and CK groups.

VOCs are key signals for communication between plants and the environment (De Moraes et al., 1998). Our data showed that methanethiol 3-hexen-1-ol and 1-penten-3-ol were significantly increased under AgNP treatment (Fig. 7). It has been reported that methanethiol could effectively scavenge reactive oxygen species in algae (Sunda et al., 2002). The accumulation of 3-hexen-1-ol was found to enhance the hyperosmotic stress tolerance of *Camellia sinensis* (Hu et al., 2020). 1-Penten-3-ol and 3-hexen-1-ol are photooxidation compounds initiated by radicals and sunlight (Jiménez et al., 2009). These results suggested that the abiotic tolerance was enhanced under AgNP treatment. Moreover, 2-hexenal, naphthalene, hexanal and 3-cyclohexen-1-ol were also found to significantly increase under AgNP treatment. At a low concentration, 2-hexenal was found to facilitate *Botrytis cinerea* infection of fruits (Xu et al., 2021). Naphthalene is a common pesticide used against moths and moth larvae (Portoni, Grau-Bové & Strlic, 2019). Hexanal was used as a quorum-sensing inhibitor to prevent diseases of Chinese cabbage and lettuce (Zhang et al., 2018b). 3-Cyclohexen-1-ol is a derivative of limonene, which is well known for its antimicrobial and antiseptic activities (Oliveira et al., 2001; Magwa et al., 2006). It is noteworthy that these VOCs can be involved in the plant response to abiotic and biotic stresses, which suggests that the cotton leaves initially produce compounds to adapt to AgNP stress. Therefore, it is of great significance to pay more attention to the impact of AgNPs on VOCs in plants.

Based on the above analyses, we proposed a metabolic map to elucidate the comprehensive mechanism of primary metabolites and VOCs in cotton leaves in response to AgNPs. The compounds significantly affected by AgNPs were scattered across the primary metabolism with the partial specific pathways involved in sugar metabolism (sucrose, xylose, arabitol) and amino acid metabolism (glycine, L-glutamic acid, alanine) (Fig. 7). Changes in primary metabolites induced by AgNPs might subsequently influence the production of VOCs via unknown pathways. In summary, this study provides a comprehensive reflection for studying the toxic effects of AgNPs on cotton. These results revealed that the changes in primary metabolites and VOCs of cotton leaves induced by AgNPs might enhance abiotic and biotic stress tolerance, but this will need to be investigated in future studies.

## Conclusions

In this study, we comparably investigated the impact of AgNPs and Ag^+^on cotton seedlings after one-week treatment. In addition to considering typical photosynthetic endpoints to evaluate the impact of AgNPs on cotton, we also evaluated the differences of primary metabolites and VOCs under AgNP and Ag^+^ treatments. While AgNPs and Ag^+^ can both induce slight physiological responses, metabolomics revealed metabolite profile alterations in cotton leaves. For the primary metabolites, a number of amide acids, sugars and sugar alcohols were altered upon exposure to either AgNPs or Ag^+^, while more metabolites were changed by Ag^+^, indicating that Ag^+^ from AgNO_3_ might be more available than that from AgNPs. VOC analysis showed more VOCs induced by AgNPs than Ag^+^, suggesting that biotic and abiotic stress tolerance might be enhanced. We demonstrated that AgNPs impacted the primary metabolic profiles, but the significantly changed VOCs might also be one of the hallmarks of AgNP exposure. This finding provides valuable information for understanding the molecular mechanisms involved in plant responses to AgNPs and will be useful for seeking sustainable protection strategies to safely control crop diseases.

## Supplemental Information

10.7717/peerj.13336/supp-1 Supplemental Information 1 Supplemental Figures and Tables

10.7717/peerj.13336/supp-2 Supplemental Information 2 Raw data

## Additional Information and Declarations

### Data Availability

The following information was supplied regarding data availability: The raw measurements are available in the Supplementary File.
