# Supplementary material for: Changes in primary metabolites and volatile organic compounds in cotton seedling leaves exposed to silver ions and silver nanoparticles revealed by metabolomic analysis
Source: PeerJ. 2022 Apr 21;10:e13336. doi: 10.7717/peerj.13336 (PMC9035277; doi:10.7717/peerj.13336)
Supplement: Supplemental Information 1 [file peerj-10-13336-s001.docx]

**Supplementary materials**

**Changes in primary metabolites and volatile organic compounds in cotton seedling leaves exposed to silver ions and silver nanoparticles revealed by metabolomic analysis**

Yong Yang, Pengmeng Du, Wenjie Lai, Liyan Yin, Yuanhao Ding, Zhonghua Li, Haiyan Hu*

Hainan Key Laboratory for Sustainable Utilization of Tropical Bioresources, college of Tropical crops, Hainan University, Haikou, 570228, China.

*Corresponding author. E-mail: huhaiyan@hainanu.edu.cn (Haiyan Hu)

**
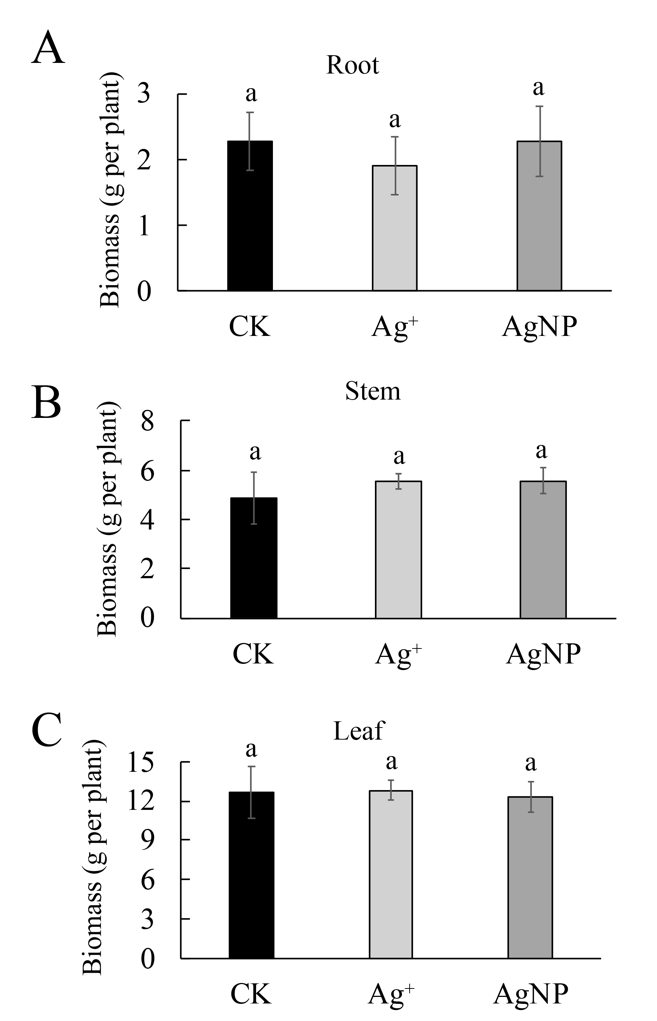
**

**Fig. S1** Biomass of root (A), stem (B) and leaf (­C) of cotton seedlings after 7 d foliar exposure to CK, Ag^+^ and AgNPs. Data are the mean of three replicates. Error bars stand for standard deviation. The letters represent statistical differences at *p* ≤ 0.05 compared with other groups.

**
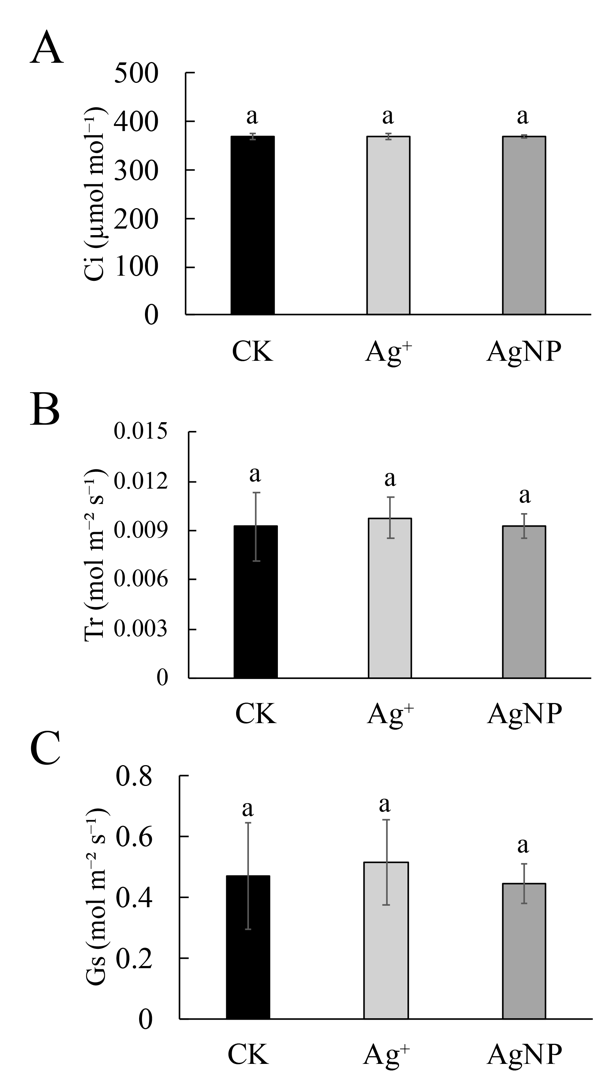
**

**Fig. S2** Photosynthetic parameters in cotton leaves. (A, B, C) intercellular CO_2_ concentration (Ci), transpiration rate (Tr) and stomatal conductance (Gs) of cotton upon exposure to CK, Ag^+^ and AgNPs.


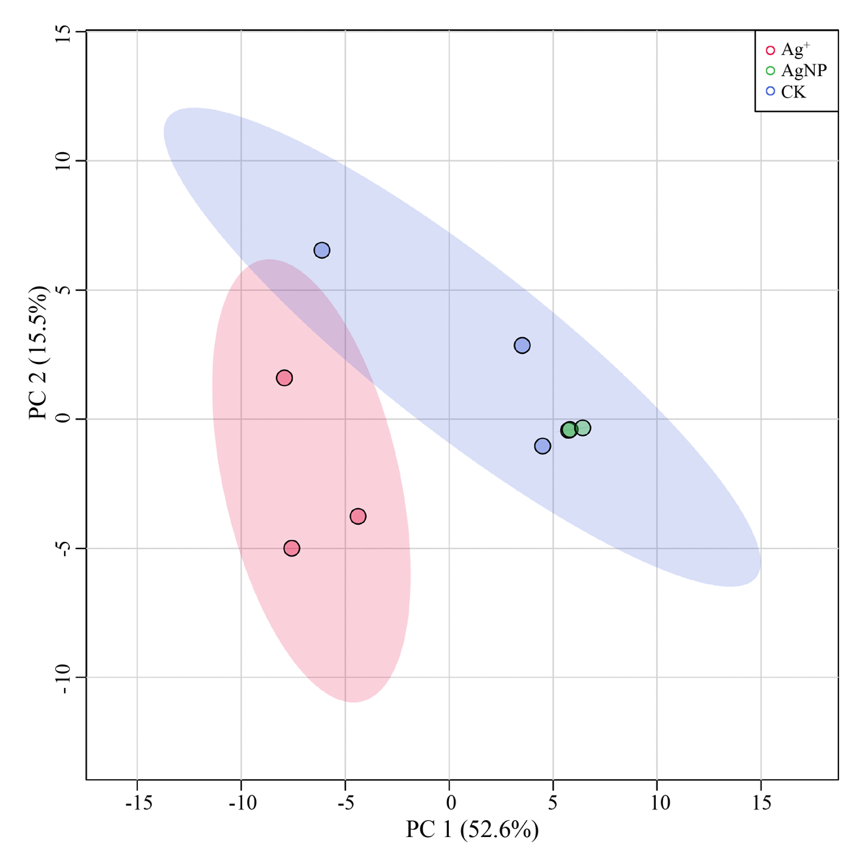


**Fig. S3** PCA score plots derived from primary metabolites extracted from CK, Ag^+^ and AgNP groups.


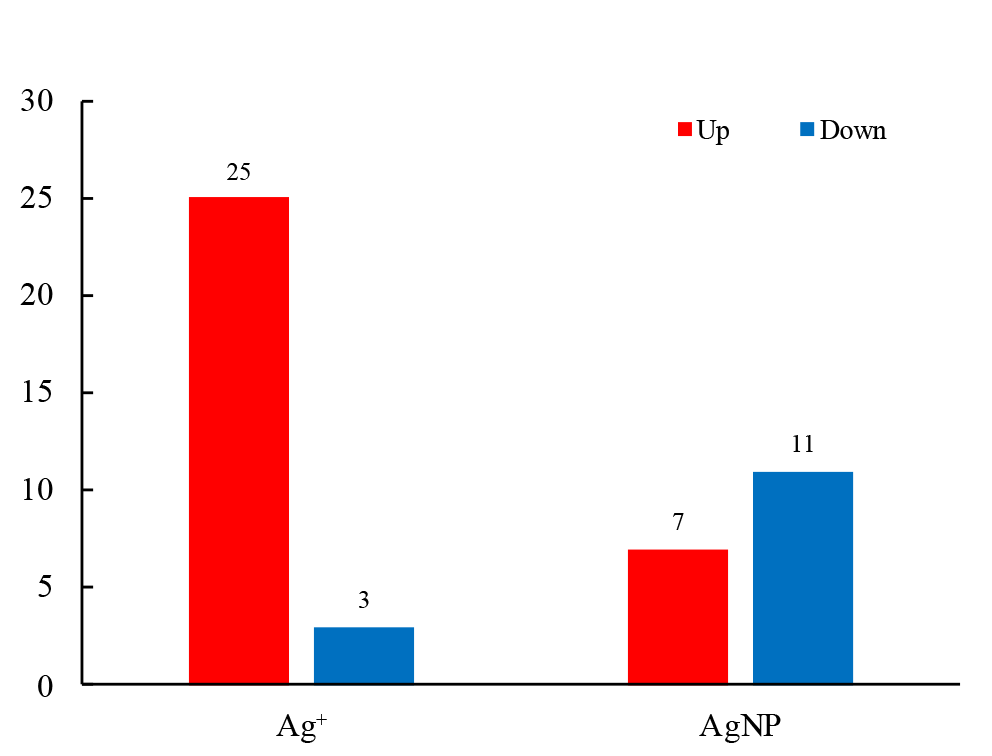


**Fig. S4** Histogram depicting the number of specific differential primary metabolites in Ag^+^ and AgNP treatment groups comparing to CK group.

**
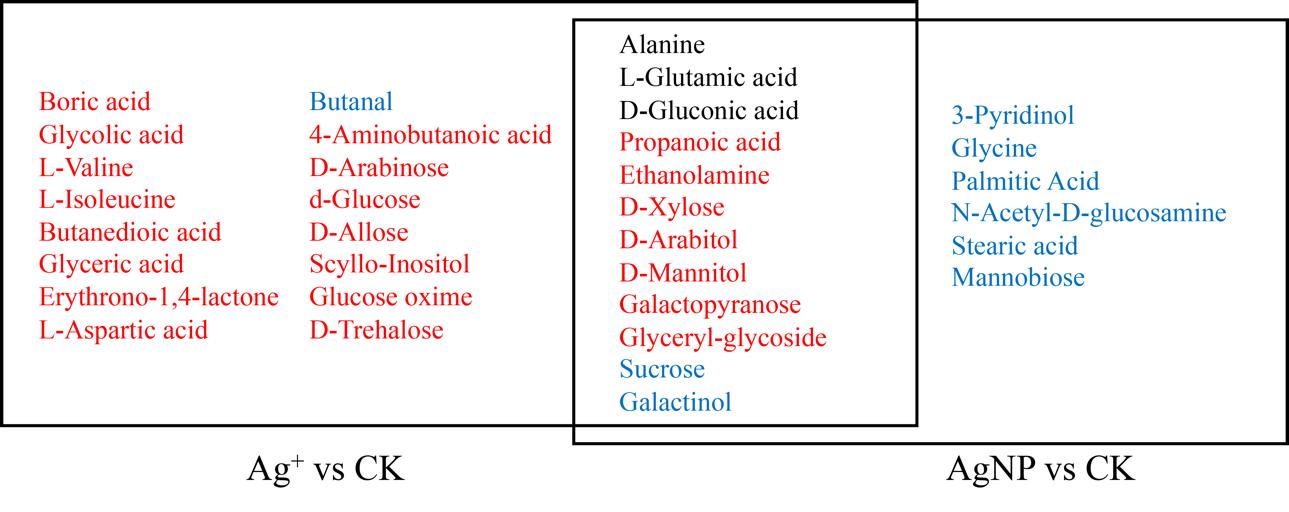
**

**Fig. S5** Venn table analysis of differential metabolites under different treatments. Red represents up-regulated, blue represents down-regulated, black represents up-regulated in Ag^+^ and down-regulated in AgNP group comparing to CK group.


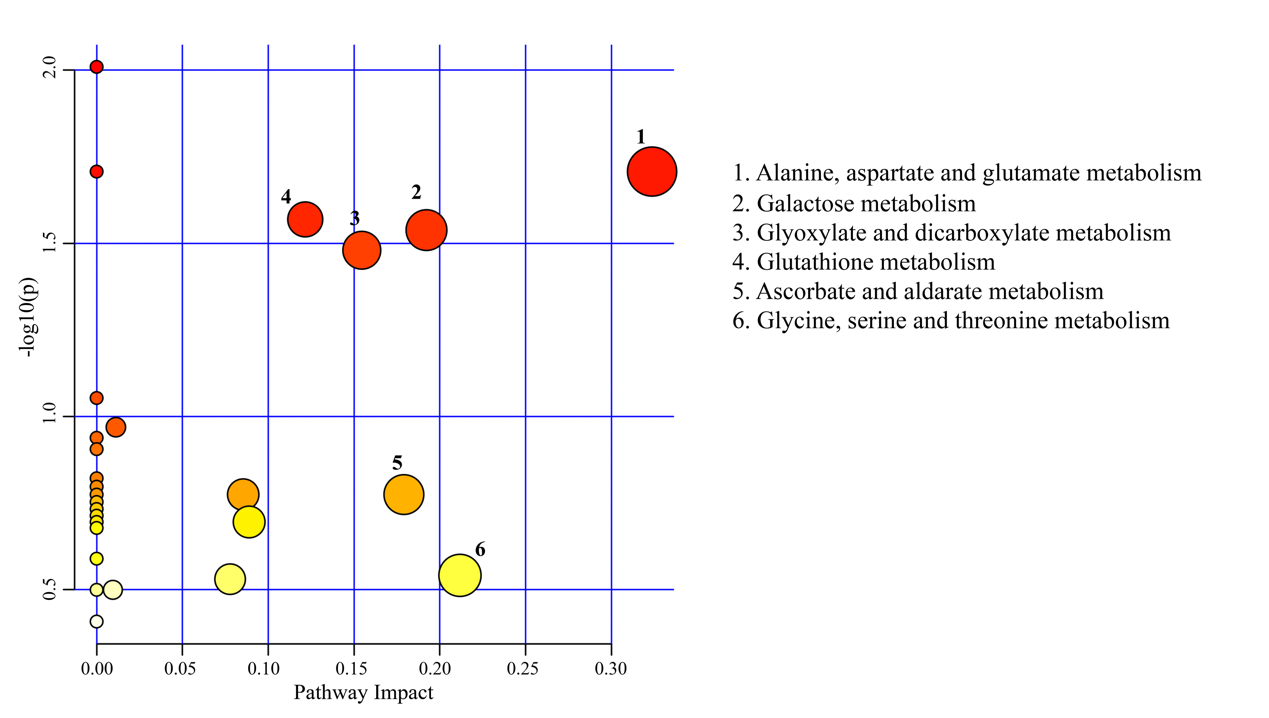


**Fig. S6** Pathway analysis of significantly differential primary metabolites in cotton leaves exposed to AgNPs.


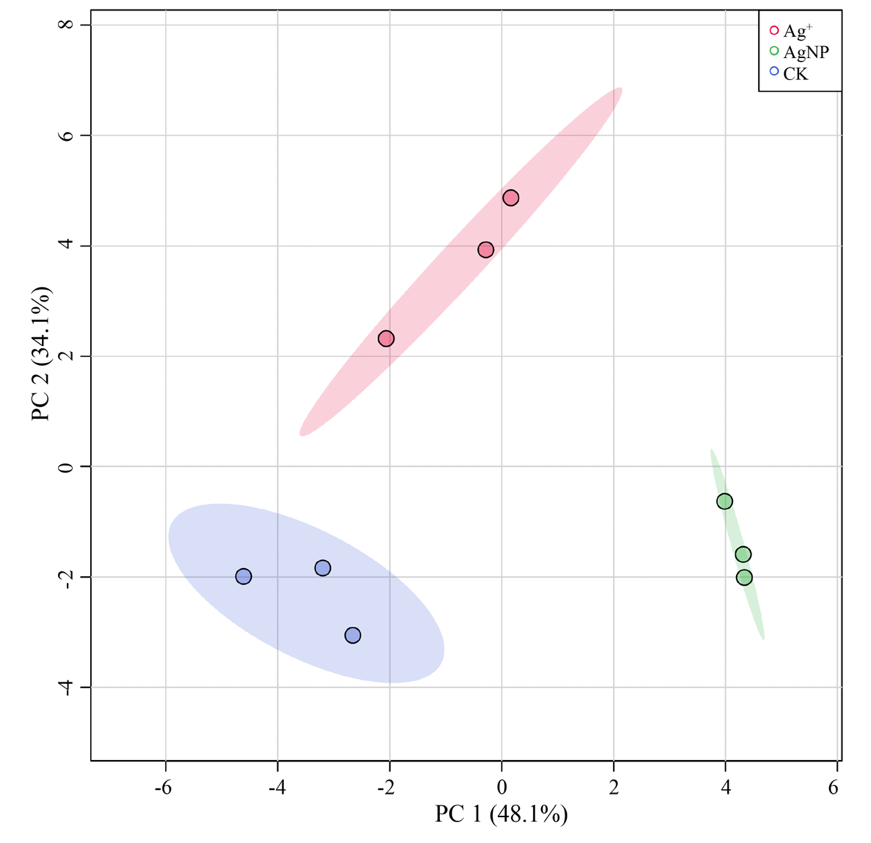


**Fig. S7** PCA score plots derived from significantly differential VOCs extracted from control, Ag^+^ and AgNP groups.

**Table S1**. The primary metabolites with their relative abundances in all the samples.

| Compounds | RT [min] | CAS | CK_1 | CK_2 | CK_3 | Ag^+^_1 | Ag^+^_2 | Ag^+^_3 | AgNPs_1 | AgNPs_2 | AgNPs_3 |
| --- | --- | --- | --- | --- | --- | --- | --- | --- | --- | --- | --- |
| Ethylene glycol | 5.488 | 7381-30-8 | 403679 | 1842131 | 1906874 | 1910355 | 1978099 | 2113582 | 1824471 | 1866155 | 1748663 |
| N,N-Dimethylglycine | 5.662 |  | 7437052 | 5296235 | 13734356 | 18589296 | 8795965 | 10615456 | 5440733 | 4268071 | 2245662 |
| Boric acid | 5.681 | 4325-85-3 | 93511 | 221112 | 634552 | 603604 | 508923 | 345664 | 220691 | 238065 | 262981 |
| 3-Pyridinol | 6.469 | 41571-88-4 | 1161160 | 3655041 | 4408547 | 4094688 | 5548434 | 6675699 | 1212711 | 1547362 | 1458076 |
| Propanoic acid | 6.798 | 55493-92-0 | 108289 | 177195 | 487401 | 777801 | 771543 | 575169 | 406972 | 494143 | 409959 |
| Lactic Acid | 6.972 | 17596-96-2 | 486142 | 910950 | 1782379 | 1039015 | 1728253 | 979033 | 509965 | 610720 | 910392 |
| Glycolic acid | 7.228 | 33581-77-0 | 263042 | 288766 | 906682 | 1111288 | 1032294 | 958987 | 242965 | 322780 | 283886 |
| L-Valine | 7.441 | 7480-78-6 | 26386 | 258835 | 695673 | 1355080 | 1661401 | 1660744 | 194047 | 218623 | 192137 |
| n-Butylamine | 7.455 | 18394-04-2 | 135719 | 580201 | 589542 | 614236 | 700409 | 1039827 | 570549 | 758976 | 795550 |
| Alanine | 7.692 | 2899-44-7 | 433960 | 566748 | 715867 | 1119028 | 2164462 | 1351517 | 256750 | 297816 | 206831 |
| Oxalic acid | 8.19 | 18294-04-7 | 470340 | 387289 | 1294135 | 1601952 | 1208291 | 1492820 | 406789 | 426523 | 233902 |
| Cadaverine | 8.499 | 65898-76-2 | 383659 | 1324459 | 1393982 | 1073076 | 1450458 | 1509623 | 661627 | 484860 | 675129 |
| L-Isoleucine | 8.9 |  | 10577 | 115073 | 259900 | 612715 | 996301 | 835356 | 106155 | 74252 | 85088 |
| Phosphoric acid | 9.045 | 18291-81-1 | 916374 | 182222 | 5008077 | 5054239 | 1455040 | 1280592 | 160575 | 254716 | 136002 |
| L-Serine | 10.138 | 70125-39-2 | 1119841 | 4610199 | 23085670 | 14139134 | 13511761 | 10262578 | 869062 | 1369494 | 1127659 |
| Ethanolamine | 10.258 | 5630-81-9 | 732704 | 744689 | 1060981 | 3546425 | 3437341 | 2867350 | 1155463 | 1457513 | 1505158 |
| L-Threonine | 10.423 | 7536-82-5 | 104369 | 698276 | 2767048 | 2596019 | 3013865 | 2615110 | 181434 | 300655 | 241456 |
| Glycine | 10.853 | 5630-82-0 | 3434275 | 1707009 | 2868272 | 2491126 | 3920923 | 3030100 | 531364 | 1070524 | 690986 |
| Butanedioic acid | 10.906 | 40309-57-7 | 931640 | 2579273 | 3858954 | 5425686 | 6911109 | 6160351 | 1969291 | 2612723 | 2238371 |
| Glyceric acid | 11.235 | 38191-87-6 | 6286306 | 4029667 | 20730196 | 30755563 | 23399256 | 19050659 | 4277737 | 5757549 | 4135004 |
| 2,5-Bis((trimethylsilyl)oxy)pyrazine | 11.559 | 362516-09-4 | 110012 | 3500 | 207182 | 226490 | 56005 | 37514 | 2870 | 11648 | 1375 |
| Erythrono-1,4-lactone | 11.815 | 55220-79-6 | 920814 | 1607915 | 2413958 | 3077990 | 3638451 | 3408145 | 441327 | 404945 | 944527 |
| Trisaminol | 12.327 | 91933-50-5 | 127771 | 46902 | 775779 | 963260 | 99174 | 104030 | 539485 | 352616 | 529322 |
| L-Aspartic acid | 12.419 |  | 6963 | 51303 | 2363907 | 1405725 | 265896 | 310637 | 15065 | 38517 | 51633 |
| Butanal | 13.323 | 56196-36-2 | 52740072 | 38910204 | 2332886 | 5592405 | 5503165 | 3585697 | 43469578 | 47741774 | 48520394 |
| Malic acid | 13.328 | 38166-11-9 | 19943431 | 10743096 | 34730336 | 40094523 | 29143445 | 26826103 | 13050791 | 16033606 | 16084238 |
| D-Threitol | 13.328 | 32381-52-5 | 204361 | 150069 | 323192 | 375294 | 545168 | 358619 | 113178 | 2538530 | 127148 |
| L-Threitol | 13.328 |  | 49052593 | 29855957 | 86547790 | 98828838 | 70724206 | 65547798 | 35257555 | 39910855 | 40749742 |
| L-5-Oxoproline | 13.719 | 30274-77-2 | 7518436 | 1893721 | 14960885 | 7368877 | 5711100 | 4753699 | 1349183 | 1749310 | 2862583 |
| 4-Aminobutanoic acid | 13.797 | 39508-23-1 | 1815391 | 1205583 | 16113534 | 6499159 | 8599593 | 2028109 | 1898953 | 2025705 | 1285581 |
| L-Glutamic acid | 13.84 | 5269-43-2 | 1777768 | 2063868 | 22206155 | 6755978 | 7004291 | 3614469 | 139056 | 347810 | 532149 |
| 2,3,4-Trihydroxybutyric acid -1 | 14.092 | 38191-88-7 | 573574 | 199557 | 1681612 | 2716929 | 1742730 | 1172835 | 182719 | 358634 | 251746 |
| Pentanedioic acid | 14.377 | 60022-87-9 | 1343027 | 1122309 | 5491701 | 6485811 | 4392170 | 3906575 | 3055254 | 2157203 | 3502509 |
| D-(+)-Ribono-1,4-lactone | 15.329 | 10589-36-3 | 1828881 | 215582 | 723429 | 1084733 | 1195953 | 1297163 | 434854 | 410862 | 309969 |
| D-(+)-Xylose | 15.469 |  | 60242 | 72803 | 148880 | 959385 | 1211553 | 817890 | 243661 | 191272 | 192259 |
| DL-Arabinose | 15.469 |  | 1727506 | 508577 | 1024665 | 1249427 | 1645977 | 1220133 | 330892 | 284515 | 270495 |
| D-Arabinose | 15.706 |  | 539389 | 226657 | 1568817 | 5990956 | 7328294 | 5113545 | 2405783 | 2093380 | 1631410 |
| D-(+)-Arabitol | 15.711 |  | 693530 | 555894 | 1089398 | 1308568 | 1793591 | 1248778 | 1801472 | 1578098 | 1212717 |
| D-(-)-Rhamnose | 16.161 |  | 210021 | 261260 | 571796 | 658936 | 1056716 | 568950 | 208529 | 229024 | 162784 |
| Ribitol | 16.775 | 32381-53-6 | 901912 | 1046004 | 2304353 | 2528611 | 3289728 | 2100862 | 12191198 | 578876 | 17486987 |
| 2,3,4,5-Tetrahydroxypentanoic acid-1,4-lactone | 16.243 |  | 1878189 | 1939639 | 4878787 | 1714087 | 2173709 | 1373009 | 784391 | 744239 | 691958 |
| Erythritol | 16.436 | 25258-02-0 | 944750 | 229336 | 2664213 | 3201161 | 2070414 | 1046432 | 210405 | 262517 | 129601 |
| Ribonic acid | 16.567 | 57197-35-0 | 111111 | 29165 | 308211 | 345044 | 266399 | 117465 | 35792 | 37097 | 17271 |
| D-(-)-Ribofuranose | 16.775 |  | 2192618 | 804472 | 1913811 | 2047351 | 2010975 | 1942726 | 614301 | 10100775 | 17504911 |
| 2-Keto-l-gluconic acid | 16.702 |  | 409507 | 92319 | 1052096 | 1512496 | 852892 | 538548 | 128144 | 145656 | 68597 |
| Fructofuranoside | 16.775 | 30788-71-7 | 7105648 | 620629 | 19146573 | 16208295 | 2344615 | 1446832 | 12206216 | 10089399 | 17456592 |
| D-(-)-Fructofuranose | 16.77 |  | 1503151 | 1315857 | 3222357 | 3979977 | 2167205 | 1766275 | 1452787 | 1097477 | 12792559 |
| Shikimic acid | 17.036 | 55520-78-0 | 1211464 | 1014887 | 3417393 | 3698567 | 4315925 | 2387155 | 1131987 | 676662 | 656202 |
| Citric acid | 17.195 | 14330-97-3 | 13445073 | 990963 | 32198716 | 17159304 | 5553653 | 3245055 | 6439143 | 10970499 | 3791511 |
| d-Galactose | 17.505 | 128705-64-6 | 8176125 | 12710301 | 18401000 | 16802202 | 21915833 | 17044379 | 3089623 | 39781277 | 2815254 |
| Quininic acid | 17.703 |  | 1220091 | 383934 | 2691919 | 2887754 | 2327501 | 1212654 | 348553 | 417521 | 207041 |
| D-Fructose | 17.838 | 56196-14-6 | 33344299 | 34510075 | 47039958 | 55451786 | 18392470 | 50266558 | 41636733 | 38841129 | 19918358 |
| d-Glucose | 18.138 | 128705-73-7 | 27567206 | 32264312 | 69107464 | 92135818 | 28964772 | 79794094 | 46521320 | 43213765 | 45219894 |
| D-Allose | 18.312 |  | 30637076 | 31137112 | 43136800 | 48456788 | 51321042 | 45701167 | 32789243 | 29946642 | 32877251 |
| D-Mannitol | 18.302 | 14317-07-8 | 1889891 | 2014376 | 3695280 | 42187562 | 6143383 | 40459047 | 30468297 | 29754375 | 30353811 |
| β-L-Mannofuranose | 18.31 | 56227-37-3 | 9600360 | 9654670 | 12646135 | 15183919 | 3488884 | 14148127 | 10033434 | 9445599 | 10195446 |
| Galactopyranose | 18.878 | 1769-00-2 | 497984 | 7323965 | 1929139 | 19992074 | 1181595 | 17603567 | 7368661 | 10606876 | 2722779 |
| D-Gluconic acid | 18.873 | 34290-52-3 | 16303244 | 12575852 | 3349026 | 4099720 | 26513488 | 24130083 | 4147000 | 5491642 | 1713035 |
| Palmitic Acid | 19.235 | 55520-89-3 | 357095 | 925917 | 928904 | 763839 | 1634225 | 1179440 | 417033 | 400587 | 376901 |
| Scyllo-Inositol | 19.385 | 14251-18-4 | 13745226 | 9077525 | 24988940 | 31020921 | 31140378 | 23994327 | 4376369 | 4647383 | 4668057 |
| Galactaric acid | 19.748 | 56272-61-8 | 430415 | 25168 | 730699 | 456402 | 208219 | 151313 | 323591 | 823674 | 147532 |
| N-Acetyl-D-glucosamine | 19.965 |  | 17919834 | 13427354 | 23210501 | 25001676 | 24298856 | 23441704 | 9271373 | 8767851 | 8221162 |
| Myo-Inositol | 19.97 | 2582-79-8 | 22016098 | 12958401 | 34422811 | 40640154 | 39693582 | 34157549 | 7829904 | 8587484 | 7291214 |
| Caffeic acid | 20.217 | 10586-03-5 | 24085709 | 807867 | 1990066 | 2872428 | 3119878 | 2162872 | 518497 | 9871667 | 555751 |
| Glucose oxime | 20.217 | 120850-89-7 | 3862834 | 2619256 | 6763227 | 10048515 | 11434431 | 7531655 | 1713754 | 1114662 | 1968190 |
| Stearic acid | 21.043 | 18748-91-9 | 154885 | 360724 | 402761 | 381114 | 605607 | 490114 | 220557 | 159291 | 182704 |
| Glyceryl-glycoside | 21.972 |  | 640788 | 447107 | 1133885 | 3567301 | 4678175 | 3054594 | 3801732 | 3662021 | 3069944 |
| D-Trehalose | 24.674 | 60065-05-6 | 27623 | 90766111 | 240144 | 115784855 | 117111318 | 109935731 | 150872 | 85530 | 3958461 |
| 3-α-Mannobiose | 23.949 |  | 612610 | 273057 | 700900 | 924160 | 1241840 | 763874 | 233908 | 124151 | 172435 |
| 5-Methyluridine | 24.079 |  | 4702802 | 9343583 | 59771493 | 4233956 | 3883675 | 3397427 | 434494 | 360356 | 340742 |
| Sucrose | 24.674 | 19159-25-2 | 156155773 | 71157163 | 151244459 | 128229038 | 133923307 | 120423981 | 2946958 | 1325875 | 3971119 |
| Maltose | 25.462 |  | 156172994 | 96002358 | 151150927 | 128249245 | 133923307 | 120309548 | 254307 | 71243 | 283943 |
| Galactinol | 27.313 |  | 1619342 | 339237 | 1245724 | 980141 | 838666 | 895582 | 87969 | 66440 | 63138 |

**Table S2**. The VOCs with their relative abundances in all the samples.

| **Compounds** | **RT** [min] | CAS | CK_1 | CK_2 | CK_3 | Ag^+^_1 | Ag^+^_2 | Ag^+^_3 | AgNPs_1 | AgNPs_2 | AgNPs_3 |
| --- | --- | --- | --- | --- | --- | --- | --- | --- | --- | --- | --- |
| Methanethiol | 1.521 | 74-93-1 | 1643657.989 | 1776501.351 | 1547055.869 | 2629755.087 | 2384160.67 | 1932009.774 | 3620232.414 | 2689200.594 | 2416340.143 |
| 3-Buten-2-ol | 2.144 | 115-18-4 | 72925680.17 | 74540735.83 | 58589776.72 | 78980728.73 | 88287938.98 | 67106022.84 | 47670039.21 | 62112595.26 | 46328108.02 |
| 1-Penten-3-ol | 2.86 | 616-25-1 | 127922265.9 | 122090305.8 | 102209730 | 184647180 | 186900878.9 | 126541377.2 | 148148179.6 | 150734849.5 | 132976371.1 |
| Pentanal | 3.066 | 110-62-3 | 247440599.6 | 210113924.9 | 180915657.4 | 241456627 | 209830003.9 | 115178422.3 | 118890371 | 128838365.8 | 118555707.7 |
| Pentane | 4.164 | 543-59-9 | 24034298.84 | 17756622.57 | 17537363.65 | 17371625.35 | 15561607.66 | 5273165.441 | 6601217.267 | 7246092.103 | 7144494.414 |
| 1-Pentanol | 4.557 | 71-41-0 | 53021862.86 | 47522338.03 | 37568780.65 | 63020758.89 | 57955820.89 | 44963209.28 | 35418999.65 | 37263997.08 | 37218091.3 |
| 2-Penten-1-ol | 4.709 | 1576-95-0 | 11139795.55 | 13274125.08 | 9405804.384 | 58194671.69 | 40426697.86 | 44913921.85 | 44211050.29 | 55552721.94 | 52470111.93 |
| Hexanal | 5.454 | 66-25-1 | 38901047.97 | 35279843.21 | 35452038.22 | 64536242.6 | 45491646.16 | 28799477.96 | 286146115.6 | 217146626 | 201827196.8 |
| Heptane | 6.175 | 2213-23-2 | 21342681.03 | 12093213.47 | 25183664.86 | 34411297.12 | 19270913.19 | 5886202.061 | 21501044.62 | 12180606.55 | 6355088.082 |
| 2-Hexenal | 7.48 | 505-57-7 | 804496.4188 | 2309906.225 | 897000.3512 | 39852252.32 | 31099706.21 | 21641683.31 | 178086950.4 | 157861640.7 | 160522290.9 |
| 3-Hexen-1-ol | 7.744 | 928-96-1 | 832416.9038 | 5962786.881 | 897000.3512 | 18010489.42 | 16529358.01 | 22967968.83 | 18112434.17 | 15584280.42 | 10326606.31 |
| Octane | 7.911 | 2216-34-4 | 20921776.83 | 10840169.48 | 18178176.6 | 24583330.33 | 11647592.99 | 2439653.764 | 12257312.54 | 6147012.45 | 58955040.73 |
| p-Xylene | 8.132 | 106-42-3 | 21372991.99 | 14245028.93 | 22701086.89 | 24751409.57 | 15174878.09 | 8246734.197 | 14833151.17 | 8553483.329 | 7806155.341 |
| Cyclohexanone | 9.226 | 108-94-1 | 71924456.66 | 68290606.69 | 64572899.65 | 24954854.42 | 24171990.93 | 19122363.59 | 64068006.14 | 62611182.73 | 63972084.26 |
| α-Pinene | 11.359 | 80-56-8 | 1015777608 | 703455242.4 | 852900310.9 | 902052542.3 | 599618026.3 | 270065806.3 | 897480337.8 | 676169720.1 | 419421334.1 |
| Camphene | 12.124 | 79-92-5 | 12516837.19 | 8808883.407 | 10232439.14 | 10769404.89 | 7476102.592 | 2717269.823 | 13004182.96 | 8551717.448 | 5094769.796 |
| Benzene | 13.08 | 611-14-3 | 94729544.85 | 76715424.74 | 27541404.24 | 101420299.3 | 71873411.22 | 38241431.8 | 52956709.78 | 41798834.98 | 32525726.41 |
| Bicyclo[3.1.0]hexane | 13.841 | 3387-41-5 | 378479419.8 | 232036393 | 260310821.1 | 265364683.7 | 137457676.9 | 75238960.26 | 246948472.3 | 166084802.2 | 112838838 |
| 5-Hepten-2-one | 14.895 | 110-93-0 | 66253537.7 | 65569359.87 | 43885749.75 | 57004776.42 | 4157965.691 | 7277044.99 | 3955331.912 | 38454369.36 | 40680187.49 |
| β-Myrcene | 15.047 | 123-35-3 | 165722240.6 | 116523766.7 | 117215230.6 | 115424395.5 | 54140822.54 | 36201203.16 | 95786854.63 | 63372801.32 | 49363511.91 |
| Decane | 15.655 | 124-18-5 | 76885097.14 | 50919938.6 | 52614580.9 | 36405554.07 | 16269751.4 | 8569182.006 | 16341225.94 | 10751286.96 | 7963740.358 |
| Limonene | 17.274 | 138-86-3 | 103700682.9 | 82057652.48 | 81868617.71 | 87599186.38 | 41570157.35 | 24040516.91 | 62972953.34 | 52135544.8 | 50107315.88 |
| 1-Hexanol | 17.71 | 104-76-7 | 29156647.06 | 42920826.61 | 29310182.06 | 35406384.14 | 33922384.76 | 47389451.81 | 27934147.38 | 43336603.25 | 41235369.04 |
| 1,3,6-Octatriene | 18.858 | 3338-55-4 | 17691470.65 | 16113887.97 | 12071639.91 | 9534237.158 | 4460996.129 | 3470124.315 | 9443590.549 | 6635129.715 | 5524128.881 |
| Undecane | 19.481 | 1120-21-4 | 31249066.34 | 27095526.87 | 32667940.01 | 49684089.11 | 19469169.53 | 14312641.82 | 14153091.15 | 8725672.137 | 8270706.323 |
| Isopinocarveol | 21.222 | 6712-79-4 | 498925.1701 | 358656.5561 | 325423.8418 | 483694.9861 | 191898.8395 | 220319.4071 | 483825.0913 | 574100.0969 | 1451043.605 |
| Nonanal | 22.742 | 124-19-6 | 16874000.81 | 31379167.02 | 24763721.9 | 42253457.92 | 22247962.17 | 20948300.7 | 7699046.728 | 5942043.081 | 6366166.293 |
| Dodecane | 29.319 | 112-40-3 | 38759086.83 | 49977800.93 | 42909333.7 | 53223553.57 | 24818770.74 | 23079498.88 | 20574077.58 | 13385916.21 | 14224680.6 |
| Benzaldehyde | 30.089 | 5779-94-2 | 82968852.58 | 85049100.06 | 31580916.3 | 98871044.42 | 106857519 | 65625672.47 | 96932443.89 | 95258905.97 | 109091243.8 |
| Bicyclo[2.2.1]heptan-2-ol | 34.797 | 5655-61-8 | 49537422.9 | 62120191.52 | 48607726.14 | 14803479.25 | 11567170.2 | 10792456.94 | 34005917.02 | 27207183 | 31611492.86 |
| γ-Elemene | 38.058 | 29873-99-2 | 5303283.879 | 7550175.037 | 5847559.991 | 3505547.567 | 2408537.271 | 2320712.9 | 6764880.354 | 5135974.641 | 6678030.378 |
| Copaene | 40.471 | 3856-25-5 | 100594915.3 | 145889296.6 | 126915176.7 | 46281268.4 | 28179180.07 | 31037961.01 | 102882275.7 | 84526211.02 | 96846149.33 |
| Caryophyllene | 43.11 | 87-44-5 | 1905236528 | 1497437155 | 1743373000 | 1383429461 | 1818423061 | 1327720320 | 1616387736 | 2100123390 | 538529424.4 |
| 10,10-Dimethyl-2,6-dimethylenebicyclo[7.2.0]undecane | 43.782 | 357414-37-0 | 86026884.39 | 57223573.43 | 52055494 | 41707379.67 | 484356603.7 | 1126341245 | 46445724.56 | 41004405.46 | 44607985.88 |
| trans-α-Bergamotene | 44.444 | 13474-59-4 | 356208570.6 | 510831663.4 | 461452042.1 | 488954355.9 | 377023167 | 342055405.5 | 488394702.8 | 456787611.3 | 486288543.3 |
| Humulene | 45.317 | 6753-98-6 | 1304142793 | 4929622197 | 4621769955 | 3779799378 | 2755123406 | 2738517692 | 4437701818 | 1568356320 | 4241211324 |
| Bicyclo[2.2.1]heptane | 45.822 | 511-59-1 | 298443081.5 | 401006938.8 | 360453591.1 | 371399043.4 | 270611631.3 | 254092320.7 | 371482754.3 | 350868919.9 | 370154239.7 |
| γ-Muurolene | 46.739 | 30021-74-0 | 10522703.13 | 14623734.65 | 12501054.51 | 9264417.589 | 7791265.722 | 6241692.098 | 14399820.49 | 14716601.39 | 15856366.29 |
| β-Bisabolene | 48.868 | 495-61-4 | 31781261.79 | 46693163.87 | 40722735.66 | 43440930.66 | 40857279.5 | 31678730.72 | 64322442.23 | 60302881.69 | 62881553.55 |
| γ-Bisabolene | 49.211 | 13062-00-5 | 925364163.4 | 1161667034 | 1049384104 | 1102288410 | 945301960.5 | 859861430.8 | 1382927928 | 1337866837 | 1366997250 |
| Naphthalene | 49.589 | 523-47-7 | 78444251.02 | 102523316.8 | 90691043.71 | 59357986.08 | 60001865.19 | 44415940.97 | 142287974.7 | 136426568 | 139130808.5 |
| Nerolidol | 52.168 | 142-50-7 | 11835194.81 | 16333308.86 | 12086871.6 | 15229362.17 | 10653918.95 | 9798516.985 | 25170085.87 | 20598083.5 | 24635690.36 |
| Caryophyllene oxide | 52.752 | 1139-30-6 | 7574581.696 | 9653692.053 | 7011800.523 | 6739093.889 | 5559563.49 | 4267833.231 | 9304385.751 | 8759436.113 | 9012245.214 |
| 3-Cyclohexen-1-ol | 58.029 | 15352-77-9 | 632456574.7 | 707573294.7 | 592725707.2 | 686754122.7 | 728526288.1 | 608762216 | 966298861.7 | 892724118.7 | 983932171.3 |
| 2,6-Dihydroxybenzoic acid | 58.593 | 3782-85-2 | 12450664.3 | 13470821.22 | 10064826.47 | 16061706.15 | 6691287.367 | 6214790.919 | 4946036.575 | 4052149.339 | 13273886.93 |
